# Mechanobiology of dynamic enzyme systems

**DOI:** 10.1063/1.5133645

**Published:** 2020-03-03

**Authors:** Peter J. Butler

**Affiliations:** Department of Biomedical Engineering The Pennsylvania State University University Park, Pennsylvania 16802, USA

## Abstract

This Perspective paper advances a hypothesis of mechanosensation by endothelial cells in which the cell is a dynamic crowded system, driven by continuous enzyme activity, that can be shifted from one non-equilibrium state to another by external force. The nature of the shift will depend on the direction, rate of change, and magnitude of the force. Whether force induces a pathophysiological or physiological change in cell biology will be determined by whether the dynamics of a cellular system can accommodate the dynamics and magnitude of the force application. The complex interplay of non-static cytoskeletal structures governs internal cellular rheology, dynamic spatial reorganization, and chemical kinetics of proteins such as integrins, and a flaccid membrane that is dynamically supported; each may constitute the necessary dynamic properties able to sense external fluid shear stress and reorganize in two and three dimensions. The resulting reorganization of enzyme systems in the cell membrane and cytoplasm may drive the cell to a new physiological state. This review focuses on endothelial cell mechanotransduction of shear stress, but may lead to new avenues of investigation of mechanobiology in general requiring new tools for interrogation of mechanobiological systems, tools that will enable the synthesis of large amounts of spatial and temporal data at the molecular, cellular, and system levels.

## INTRODUCTION

Blood flow induces shear stress on the endothelial cell surface and initiates a series of biochemical pathways.^1–4^ For example, shear leads to the generation of nitric oxide (NO), a potent vasodilator in small arterioles, and a potent inhibitor of platelet adhesion and aggregation.^5–9^ The formation of nitric oxide, therefore, has intrigued scientists studying both control of blood perfusion of the microvasculature and the pathobiology of atherosclerosis and thrombosis in larger arteries.^6,10^ Shear stress also activates the mitogen activated protein kinase (MAPK) signaling pathway, leading to inductions of the promoters of monocyte chemotactic protein-1 (MCP-1) and c-fos, molecules that facilitate early monocyte recruitment to fatty lesions in arteries.^11^ Shear induction of nitric oxide^12^ and the MCP-1 and c-fos promotors^13^ depended on transmembrane integrin molecules.

**FIG. 1.**
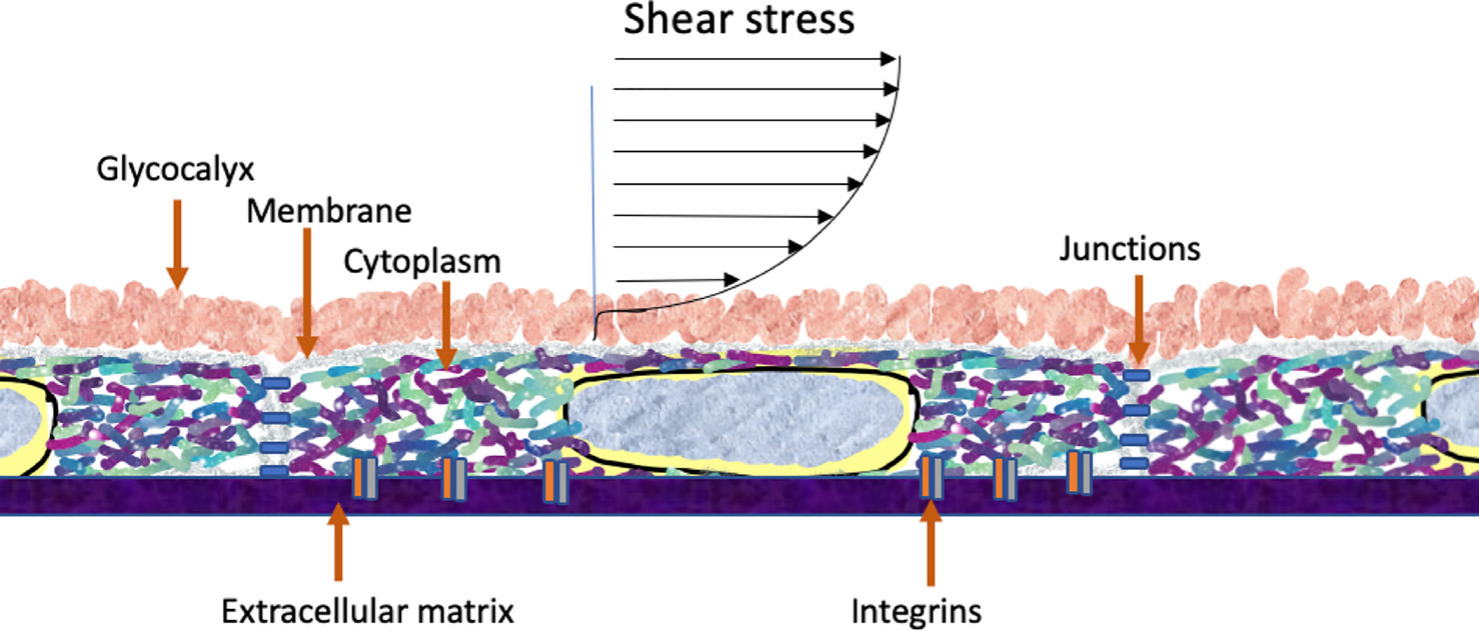
Shear stress of vascular endothelium. Figure denotes the crowded nature of the glycocalyx, membrane, and cytoplasm. These structures are inherently dynamic and their dynamic structures are sensitive to the prevailing shear stress. The structures are also arenas for enzyme-driven signaling pathways, the location and kinetics of which are also dependent on applied force.

The convergence of research into the fundamental mechanisms by which endothelial cells convert mechanical force into biochemical signaling has been a central focus of the lab of Shu Chien. This review traces two avenues of investigation championed by the Chien lab: integrin mechanosensation and the mechanobiology of the cell as a complex, interconnected, dynamic system. The review will lead to a hypothesis that cellular systems from single integrin molecules to two-dimensional organization of membrane molecules to three-dimensional readjustment of cytoskeleton-driven rheology and organization of enzymatic signaling systems constitute mechanotransduction pathways manifested in force-dependent changes in endothelial cell biology. Specifically, a new line of research in mechanobiology is proposed that focuses on the crowded environment of the cytoplasm in three dimensions and membrane in two dimensions. These cellular structures are arenas for enzymatic pathways and may be driven by force to various non-equilibrium states, resulting in observed changes in cellular behavior (Fig. 1). Put another way, the cell as a dynamic and integrated engineering and biochemical system is mechanosensitive.^14^

## ENDOTHELIAL CELLS ARE DIFFERENTIALLY SENSITIVE TO SHEAR MAGNITUDE AND RATE-OF-CHANGE

Shear stress magnitude and rate-of-change result in dramatically different endothelial cell-mediated vasodilations.^15–17^ Shear-induced vasodilation depends on integrins,^18,19^ cell-cell junctions,^6^ membrane channels,^20^ glycocalyx,^21^ and microtubules,^10^ among other components. Thus, it is likely that the rate sensitivity arises from the matching of time constants between the stimulus (force) and the dynamic organization of these force-sensitive systems.^22^ The rate sensitivity of endothelial cells in larger arteries had been suspected for many years and has led to a series of discoveries that shear rate-of-change may be a more potent stimulus to cells than the shear magnitude itself.^23–25^ In fact, the leading hypothesis for the localization of atherosclerotic lesions is that shear stress in areas of disturbed flow, which is caused by sudden changes in shape and curvature of blood vessels, occurs where shear stress is low and oscillatory, whereas shear stress in areas of the vasculature protected from atherosclerosis is unidirectional and relatively high.^26^ Thus, endothelial cells of all sized blood vessels are sensitive to the shear magnitude, direction, and rate-of-change.

## THE MEMBRANE'S CONSTITUENTS ARE DYNAMICALLY REARRANGED BY SHEAR STRESS

The search for a mechanosensor that is sensitive to rates and magnitudes of shear application led to the hypothesis that the membrane can transduce force to biochemical signals.^27–29^ The cell membrane has relatively strong structural integrity in the lateral dimension but has a fairly low bending modulus, making it simultaneously a formidable structure that helps define the cell boundary, and a dynamic structure capable of rapid sensation of external forces.^30^ It has subsequently been shown that shear stress applied to the luminal endothelial cell surface leads to increases in the dynamic reorganization of membrane lipids^29,31,32^ and that this change in lipid fluidity is felt in membrane subdomains.^33^ It has further been shown that shear leads to out of plane membrane undulations,^34^ which could play a role in cellular interaction with the environment.^30^ These changes were seen to depend on the shear direction as evidenced by the finding that an increase in the lateral diffusion of a lipid-like dye was higher in the upstream facing part of the cell and lower in areas downstream of the nucleus.^32^ Furthermore, shear-induced changes in membrane fluidity depended on the rate of change of shear stress.^31^ Thus, the cell membrane was uniquely suited to be a mechanosensor of shear stress that could detect direction, magnitude, and rate of change, features of shear stress now known to be primary determinants of the location and formation of atherosclerotic lesions.

Focus on the membrane as a heterogenous system with regulated spatial organization of membrane molecules^35^ has led to a hypothesis that shear stress may be uniquely felt in membrane microdomains. For example, a simplifying concept has emerged that characterizes cell membranes as existing in liquid-ordered (L_o_) and liquid disordered (L_d_) domains. L_o_ domains are high in cholesterol and characteristically thicker and more viscous^36^ than L_d_ domains. Accordingly, L_o_ domains were labeled using long-chain DiI molecules (DiI C_18_), whose fluorescence lifetime is higher owing to the viscous nature of surrounding molecules. Conversely, short-chain DiI molecules (e.g., DiI C_12_) associate with L_d_ domains and exhibit short fluorescence lifetimes. In studies on shear effects on membrane microdomains, shear stress leads to fluidization of the L_d_ domains in a rapid and transient fashion while the increase in L_o_ domains was slower and more sustained. This result may be explained by the idea the L_d_ domains have a lower modulus than liquid ordered domains.^37^ The lower modulus makes them rapidly sensitive to changes in luminal shear. Alternative methods using dyes sensitive to water penetration of the membrane have yielded similar conclusions.^38^ Once sheared, after an initial transient increase in fluidity, domains are likely to coalesce.^39^ Taken together, it is possible that shear stress drives the association of domain-associated lipids and proteins from one state to another thus driving the association (and disassociation) of these molecules to a new steady state, and possibly to a new biological signaling state.

Possible membrane bound proteins responsible for shear sensitivity were receptor tyrosine kinases (RTKs); growth factor receptors that, when dimerized, lead to activation of p21 ras, an important small GTPase. The Chien lab showed that shear stress induced the activation of small GTPase p21 ras that, in turn, led to initiation of the mitogen activated protein kinase (MAPK) signaling cascade leading through c-Jun N-terminal kinase (JNK) phosphorylation and the initiation of the MCP-1 protein expression. MCP-1 is largely responsible for the adhesion of monocytes to vascular endothelial in areas of disturbed flow. Monocytes in turn migrate across the endothelium to engulf low density lipoprotein, leading to the formation of foam cells and resulting in fatty streaks.^40^ Thus, the small GTPase, which is associated with the cell membrane, is an important upstream singling partner for shear-induced activation of genetic transcription. Activation of this pathway depends on src family kinases,^11^ which may originate in L_o_ domains.^41^

The Chien group later showed convergence of RTKs and integrin signaling.^42^ In this study, they investigated the interplay of integrins, and the vascular endothelial growth factor receptor 2 (Flk-1). Flk-1 is a receptor tyrosine kinase that, when activated by vascular endothelial growth factor, dimerizes leading to regulation of MAPK and subsequently to the NF-KB pathways via Rho and Ras GTPases. Shear stress activates both the Flk-1 and integrins suggesting that shear activates multiple pathways that converge to elicit the cellular response such as alterations in gene transcription. In that study, they showed that integrins are required for the shear stress activation of Flk-1 putting integrins upstream of shear activation of their receptor tyrosine kinase. Thus, this research showed the primary role of integrins in shear sensitivity of the endothelium.

## SHEAR STRESS ON THE LUMINAL SIDE LEADS TO FORCE TRANSMISSION AND DYNAMIC RESTRUCTURING OF THE ABLUMINAL SIDE OF THE CELL

A controversy in the field was whether shear stress was felt at the luminal cell surface where it is transduced into biochemical signals, or whether the forces were transmitted to the interior^43,44^ or the basal, abluminal part of the cell.^45^ While integrins are found in both abluminal and luminal parts of the cell, the Chien lab demonstrated that new integrin adhesion with extracellular matrix molecules was responsible for shear induced MAPK activity.^13^ In this study, they plated endothelial cells on vitronectin and fibronectin and blocked new integrin ligation using antibodies that recognize the conformation of integrins unique to binding. The significance of this finding is in the recognition that forces from shear may, first, be transmitted to the abluminal side of the cell and that the forces need not be so high to actually pull on integrins and change their activation state, but rather, slight deformation of the cell can lead to new integrin ligation. Thus, high shear stress, which is atheroprotective, may be sufficient to activate integrins already bound to extracellular matrix molecules whereas lower shear may be sufficient to deform the peri-focal adhesion membrane leading to new ligation, which could then lead to the downstream signaling thought to be responsible for the formation of adhesion molecules, such as MCP-1, on the cell surface and atherogenesis.

In a recent study, preliminary quantitative support for this idea was provided using finite element analysis of sheared and focally adhered endothelial cells wherein forces from luminal shear stress are transferred through the cell to areas of focal adhesions, where they are amplified.^46–48^ This amplification comes from the juxtaposition of adhered sections of the membrane with non-adhered, resulting in relatively high strains in the peri-focal adhesion areas. Furthermore, there is evidence that the moderate shear stress is sufficient to bend the membrane toward the extracellular matrix.^49^ Further computational analysis suggested that shear stress can transmit forces to cell junctions and to the focal adhesion plaques themselves to a level sufficient to support the hypothesis that bound integrins may also be sensitive to force.^47,48^ Such force transmission may be sufficient to alter interactions of integrin and talin, a known indicator of integrin activation.^50^

Following the apposition of the membrane to extracellular matrix, it has been shown directly that this process can lead to integrin adhesion followed by activation. Using a technique based on ion conductance spectroscopy, a small pipette, functionalized with fibronectin, was brought into close proximity to the endothelial cell surface.^51^ Simultaneous imaging of fluorescently tagged GPI-anchored proteins (indicators of L_o_ lipid rafts) and RFP talin, an indicator of integrin activation, demonstrated that one of the earliest events upon adhesion is assembly of liquid-ordered domains at the point of adhesion.^52^ These domains likely recruit integrin molecules to them which would explain the arrival or talin within 10–20 s after adhesion. Thus, new adhesions between the membrane and extracellular matrix (ECM) lead to integrin transport and activation resulting in talin assembly.^53^

## INTEGRINS DIFFUSE IN THE PLANE OF THE MEMBRANE AND CONVERT FROM UNBOUND TO BOUND STATE WITH CONCURRENT MOVEMENT FROM L_d_ to L_o_ DOMAINS

 Integrin transport and adhesion was measured directly using an optically trapped micron-sized bead functionalized with Arginine, Glycine, and Aspartate (RGD) extracellular matrix molecules.^54^ Unique to this experimental setup was the ability to determine the precise moment of adhesion by detecting the refraction of light that occurs between the trapped bead and cell surface. This event was manifested as a 3% reduction in light detected using a quadrant photodiode that also functioned to determine the lateral spatial location of the bead relative the trapping laser beam. In these experiments, both the adhesion time of the bead to the surface and subsequent force production were measured. It was found that 0.5 s adhesion time resulted in high probability of single bond formation between $\beta_{1}$-integrins and the RGD peptides. When the membrane was manipulated with the amphiphile, benzyl alcohol (BA), which has been shown previously to result in reduction in L_d_ membrane thickness and the subsequent coalescence of membrane domains,^55^ affinity of the integrin to the bead increased. Integrin affinity was measured by changing the rate of retraction of the bead from the cell. Affinity was also manifested by an increase in integrin-RGD bond strength from 30 pN to 40 pN, reflecting that these are catch bonds as demonstrated in Ref. 56 Furthermore, BA's ability to cause coalescence of domains enabled testing of the hypothesis that integrin avidity would also increase as domains brought integrins closer to the contact point. Indeed, when increasing the bead-membrane apposition to 1.5 s, from 0.5 s, the number of bonds increased to two from one, but only when the cell was treated with BA.

The notion of integrin clustering in the membrane prior to adhesion was supported by using red fluorescent protein- (RFP-) labeled integrins and measuring their diffusion and brightness before and after BA treatment. Using fluorescence correlation spectroscopy, which provides two independent measures of number of molecules diffusing past a focused confocal laser beam, it was shown that integrins diffused as pairs after BA treatment. This coalescence of domains and integrins was reversed by vitamin E, which disperses domains. Domain coalescence and disbursement were verified by performing fluorescence correlation spectroscopy (FCS) analysis on Lyn, a src family kinase, known to associate with L_o_ domains.^57^ The dynamic sorting of membrane domains was shown by others using stimulated emission depletion (STED) microscopy that the natural time scale of organization of L_o_ domains^58^ was tens of milliseconds with a length scale of 10–20 nm,^59^ and dependent on adhesion to the extracellular matrix.^51^

**FIG. 2.**
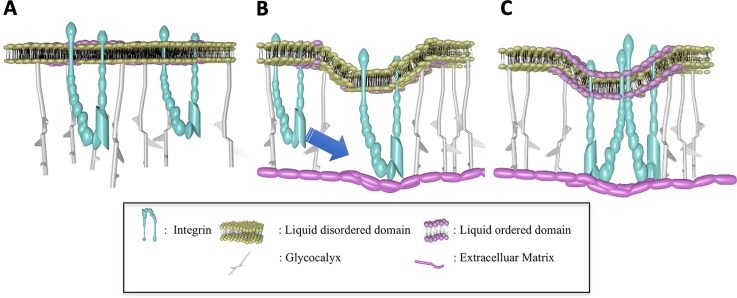
Integrin adhesion at sites devoid of glycocalyx with integrin catch bonds supported by elasticity of glycocalyx. A. Integrins exist as monomers in liquid-ordered domains with glycocalyx present uniformly on the cell surface. B. Random defects (or induced defects) in the glycocalyx spacing, thermally induced bending of the membrane, and applied force on the abluminal surface, bias the membrane to close approximation with extracellular matrix molecules. C. Once an integrin molecule is adhered, the bent membrane provides an avenue for additional adhesion, with the glycocalyx providing some of the counter forces of adhesion necessary to maintain catch bonds.

Therefore, it is suggested that shear stress on the luminal side can lead to activation of integrins on the abluminal side and their coalescence, a process related to integrin localization in membrane microdomains. Shear further assists in biasing the abluminal structure toward new adhesions by assisting in presenting integrins to the ECM. The focal nature of these adhesions is ensured because of the repelling nature of the glycocalyx, which is necessary for the formation of the second integrin bond when presenting the RGD-coated bead to the cell for 1.5 s.^54^ This concept that focal adhesions depend on the glycocalyx has been advanced recently by others.^60,61^ This concept is illustrated in Fig. 2.

## ENZYME CATALYSIS IS A NEW FORCE IN MECHANOBIOLOGY

Both the membrane and cytoplasm are arenas for enzymatic processes in which enzymes interact with ligands leading to the production of substrate molecules. Recently, it has been found that, like kinesins and other cytoskeletal bound motor proteins, diffusing enzymes convert the substrate to products and produce a “kick” force that enhances the diffusion of the enzyme.^62^ In that study, fluorescence correlation spectroscopy (FCS) was used to measure single molecule diffusion of urease, in the absence and presence of urea, and in the absence and presence of an inhibitor. It was found that urea caused a dose dependent increase in diffusion. Using Langevin molecular dynamics simulations in which a force was applied for each enzymatic turnover event until the diffusion coefficient matched that which was measured using FCS, this force was estimated to be about 12 pN, which is on the order of the force exerted by the well-known molecular motors of dynein, kinesin, and myosin.^63^ Such a discovery pointed to a series of studies that showed that when normally diffusive enzymes were bound to a substrate, they could impart forces on the local fluid and drive fluid flow.^64^ Furthermore, membrane bound enzymes such as ATPase could impart forces to the membrane themselves and cause motion of nano-sized vesicles^65^ a phenomenon that, in cells, could be responsible for non-thermal membrane bending fluctuations.^34,66,67^ Importantly, this force may be responsible for the observation that enzymes chemotax up substrate concentration gradients, which was detected using a two-channel microfluidic device.^68^ In these experiments, the substrate was introduced into a flow stream alongside the corresponding enzyme and the enzyme was seen to diffuse toward the substrate channel to a greater degree when the substrate was present than when absent or in the presence of an inhibitor. Such a phenomenon was hypothesized to be responsible for the observation of the transient existence of purinosome-related signaling complexes first observed in 2008 in which two enzymes catalyzed four sequential biochemical steps that were dynamically regulated using purine concentration.^69^ Recently, it has been suggested that the origin of this compartmentalization of signaling may be a combination of enzyme related forces, single molecule chemotaxis, and enzyme channeling.^70^

## CONVERGENCE OF ENZYMATIC MOTORS AND MECHANOTRANSDUCTION PATHWAYS: FORCE-DEPENDENT, DYNAMIC ORGANIZATION OF SUBCELLULAR SIGNALING COMPLEXES

Research described in this review has focused on force transduction mechanisms centered around the cell surface, which is made of glycocalyx, membrane, and cortical cytoskeleton. Similarly, the internal part of the cell is dynamically sensitive to shear stress as evident by fluidization (i.e., reduction of rheological moduli) of the cytoskeleton upon shear application.^71^ Such fluidization of the interior of the cell has been documented for other types of force applications.^72–77^ This fluidization points to dynamical resetting of cellular mechanics on the same time scale as application of many of the physiologically relevant mechanical stimuli. In an excellent review on the importance of the matching of time constants between stimuli and response, Hoffman and colleagues point out that mechanobiological processes can proceed very differently depending on the current state of the cell and the timing and magnitude of the stimulus.^22^ With a focus specifically on the molecular fluidization, this process suggests a dynamically evolving crowded landscape for molecular processes upon force stimulation. Interestingly, crowding can affect how enzymes are transported through cell-like environments.^78^ Together with the observation that diffusive enzymes can exert force to enhance their own diffusion,^79^ allowing them to travel up substrate concentration gradients^80^ and resulting in biologically relevant organization and coalescence of signaling molecules whose products are substrates for other enzymes,^70^ it is possible that endogenous enzymatic pathways may be sensitive to force through fluidization of the compartments in which they react. The concept of enzymatic forces driving biological signaling in a diffusive environment that is mechanically dynamic may mean that the cell is constantly in a state of flux, with its homeostasis dependent on well-defined, regular force stimulation.^79,80^ Such dynamical resetting of homeostasis of the cell could explain why unidirectional shear stress results in atheroprotective cells while oscillatory, dynamically reorienting shear leads to endothelium that is atheroprone.^81^ This concept of shear stress and cellular homeostasis was predicted by Shu Chien and explained in a the 2006 Cannon Award Lecture entitled “Mechanotransduction and endothelial cell homeostasis: the wisdom of the cell,”^2^ a publication that honors Dr. Chien's academic grandfather, Walter B. Cannon.^82^

## THE FUTURE OF MECHANOBIOLOGY IS IN FORCE-SENSITIVITY OF DYNAMIC SYSTEMS

**FIG. 3.**
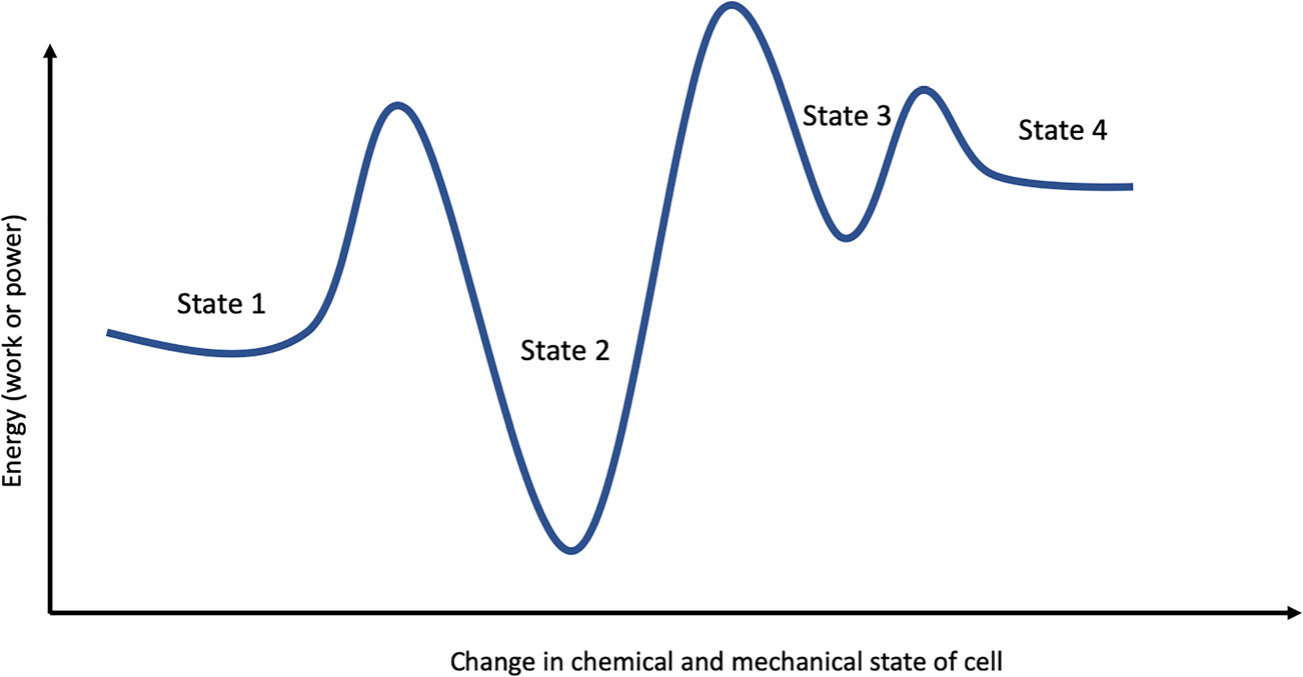
Force-dependent transitions of cellular structural and biochemical states. The diagram depicts the concept that force application (with its dynamic and magnitude components) can assist the cell in transitioning from one non-equilibrium state to another. Each of these states may constitute a distinct biological phenotype for the cell.

The existence of enzymatic forces in cells could point to a whole new concept of mechanobiology. By virtue of being in a nutrient environment, the cell is constantly metabolizing numerous substrates. Each of these enzymatic reactions may generate the force driving the mechanical and chemical fluctuations of the system in a non-equilibrium manner. In the absence of external stimuli, the non-equilibrium state may be metastable but could be driven to a new non-equilibrium state by applying force (Fig. 3). Furthermore, many enzymatic pathways in the cells comprise kinases, which catalyze the conversion of phosphate bound purines (e.g., ATP and GTP), which has been shown recently to be force producing.^65^ Conversely, depletion of ATP leads to a sharp reduction in motion of organelles in the cell.^83^

The tools to explore this area of mechanobiology may already exist. Super-resolution microscopy is rapidly improving its ability to track dynamic systems.^84,85^ Large scale imaging remains a challenge at these length and time scales but would be required to map the emergent patterns of mechanosomal organization. Furthermore, a suggested next avenue in mechanobiology research is to develop computational tools that take into account the dynamic structural/biochemical landscape that is the cell. Perhaps these tools are less mechanistic and more rules-based such that they could be assessed using neural networks, genetic algorithms, or an artificial intelligence framework, the models of which could be trained on cellular mechanobiological data, as is being done in some immunological studies,^86^ or tools from researchers focused on active matter.^87^ Since cells dynamically change the density of the cytoskeleton^71^ and crowding controls the diffusion of flexible molecules,^78^ perhaps shear stress works indirectly on signaling complexes through its dynamic control of intracellular molecular crowding. Such processes could operate in two dimensions (such as the cell membrane) or in three dimensions in the cytoplasm. Such approaches could uncover unknown parameters that pinpoint the relationship between force and dynamic mechanosome organization.^88–90^

## SHU CHIEN: PUSHING THE BOUNDARIES OF BIOLOGY AND MECHANICS

The fundamental mechanisms underlying pathophysiological processes lie at the intersection of chemistry, biology, and mechanics. Chemistry describes the reactants and reaction rates central to biological signaling; biological structures control spatial and time-dependent organization of reactions; and force influences reaction rates, structural integrity, and subcellular organization. At this intersection is mechanobiology, a field employing a diverse set of tools and scientific mindsets to understand and cure disease and develop new diagnostic and therapeutic tools. Here, we focus on the mechanobiology of the endothelium and, specifically, the cellular interface connecting extracellular matrix, glycocalyx, membrane, integrins, and cytoskeleton. As part of this special edition honoring Shu Chien, we trace a line of research originating from rate sensitivity of shear-induced arteriolar vasodilation through dynamic organizing principles of the endothelial cell membrane and integrin-mediated adhesion, and how this dynamic organization is tuned to the prevailing dynamics and magnitude of applied force. A parallel body of research is described that originates from a discovery that diffusing enzymes exert force on the surrounding fluid for each catalyzed reaction, forces that result in molecular chemotaxis up substrate concentration gradients and dynamic organization of enzyme signaling complexes. We then propose a new perspective in mechanobiology: that force drives the cell from one non-equilibrium chemical and structural state to another. This new framework moves away from deterministic causes and effects to a concept of the cell as an integrated, dynamic engineering system sensitive to its mechanical surroundings.

This review focused on how early work in the Chien lab inspired research aimed at elucidating mechanobiological mechanisms originating from the cell membrane and its integrated structures including the glycocalyx, cytoskeleton, and transmembrane proteins. Despite the search by many, the path has not so much converged on the identity of a mechanosensor as on the realization that mechanosensing may depend on dynamics and spatial complexity of the force application and the cellular structure and signaling cascades. It seems likely, therefore, that the answer to how cells sense force is less like the traditional lock and key hypothesis of receptor-ligand interaction leading to biochemical signaling, and more akin to how organisms, or groups of organisms, respond in a coordinated fashion to external stimuli. The rules of this response remain to be worked out, but it seems that the tools likely exist at interdisciplinary boundaries between enzyme kinetics, cellular structure, super-resolution microscopy, and computational science. Pushing on these boundaries is likely to lead to a more comprehensive picture of mechanobiology.

## References

[1] D. D. E. Ingber , FASEB J. 20, 811 (2006). 10.1096/fj.05-5424rev 16675838

[2] S. Chien , *Mechanotransduction and Endothelial Cell Homeostasis: The Wisdom of the Cell* ( Am J Physiol Heart Circ Physiol., 2007). 10.1152/ajpheart.01047.2006 17098825

[3] Y.-S. J. Li , J. H. Haga , and S. Chien , J. Biomech. 38, 1949 (2005). 10.1016/j.jbiomech.2004.09.030 16084198

[4] J. Y.-J. Shyy and S. Chien , Circ. Res. 91, 769 (2002). 10.1161/01.RES.0000038487.19924.18 12411390

[5] I. Fleming and R. Busse , Cardiovasc. Res. 43, 532 (1999). 10.1016/S0008-6363(99)00094-2 10690325

[6] Z. Bagi , J. A. Frangos , J.-C. Yeh , C. R. White , G. Kaley , and A. Koller , Arterioscler., Thromb., Vasc. Biol. 25, 1590 (2005). 10.1161/01.ATV.0000170136.71970.5f 15890968

[7] J. A. Frangos , T. Y. Huang , and C. B. Clark , Biochem Biophys Res Commun. 224, 660 (1996). 10.1006/bbrc.1996.1081 8713104

[8] Y. S. Chang , J. a Yaccino , S. Lakshminarayanan , J. a Frangos , and J. M. Tarbell , Arterioscler., Thromb., Vasc. Biol. 20, 35 (2000). 10.1161/01.ATV.20.1.35 10634798

[9] G. K. Kolluru , S. Sinha , S. Majumder , A. Muley , J. H. Siamwala , R. Gupta , and S. Chatterjee , Nitric Oxide 22, 304 (2010). 10.1016/j.niox.2010.02.004 20188204

[10] D. Sun , a Huang , S. Sharma , a Koller , and G. Kaley , Am. J. Physiol. Heart Circ. Physiol. 280, H2087 (2001). 10.1152/ajpheart.2001.280.5.H2087 11299210

[11] S. Jalali , Y. S. Li , M. Sotoudeh , S. Yuan , S. Li , S. Chien , and J. Y. Shyy , Arterioscler., Thromb., Vasc. Biol. 18, 227 (1998). 10.1161/01.ATV.18.2.227 9484987

[12] J. M. Muller , W. M. Chilian , and M. J. Davis , Circ. Res. 80, 320 (1997). 10.1161/01.RES.80.3.320 9048651

[13] S. Jalali , M. A. del Pozo , K. Chen , H. Miao , Y. Li , M. A. Schwartz , J. Y. Shyy , and S. Chien , Proc. Natl. Acad. Sci. U. S. A. 98, 1042 (2001). 10.1073/pnas.98.3.1042 11158591 PMC14705

[14] S. Son and P. J. Butler , Biomed. Eng. Lett. 5, 172 (2015). 10.1007/s13534-015-0198-7

[15] P. J. Butler , S. Weinbaum , S. Chien , and D. E. Lemons , Microcirculation 7, 53 (2000). 10.1111/j.1549-8719.2000.tb00742.x 10708337

[16] K. E. Pyke , J. A. Hartnett , and M. E. Tschakovsky , J. Appl. Physiol. 105, 282 (2008). 10.1152/japplphysiol.01190.2007 18467554

[17] K. E. Pyke , E. M. Dwyer , and M. E. Tschakovsky , J. Appl. Physiol. 97(2), 499–508 (2004). 10.1152/japplphysiol.01245.2003 15064302

[18] L. Loufrani , K. Retailleau , A. Bocquet , O. Dumont , K. Danker , H. Louis , P. Lacolley , and D. Henrion , Am. J. Physiol. Circ. Physiol. 294, H1906 (2008). 10.1152/ajpheart.00966.2006 18245559

[19] M. D. Frame , R. J. Rivers , O. Altland , and S. Cameron , J. Appl. Physiol. 102, 2279 (2007). 10.1152/japplphysiol.00537.2006 17379749

[20] S. A. Mendoza , J. Fang , D. D. Gutterman , D. A. Wilcox , A. H. Bubolz , R. Li , M. Suzuki , and D. X. Zhang , Am. J. Physiol. Heart Circ. Physiol. 298, H466 (2010). 10.1152/ajpheart.00854.2009 19966050 PMC2822567

[21] U. Pohl , K. Herlan , A. Huang , and E. Bassenge , Am. J. Physiol. 261, H2016 (1991). 10.1152/ajpheart.1991.261.6.H2016 1721502

[22] B. D. Hoffman , C. Grashoff , and M. A. Schwartz , Nature 475, 316 (2011). 10.1038/nature10316 21776077 PMC6449687

[23] X. Bao , C. B. Clark , and J. a Frangos , Am. J. Physiol. Heart Circ. Physiol. 278, H1598 (2000). 10.1152/ajpheart.2000.278.5.H1598 10775139

[24] X. Bao , C. Lu , and J. A. Frangos , Am. J. Physiol. Heart Circ. Physiol. 281, H22 (2001). 10.1152/ajpheart.2001.281.1.H22 11406464

[25] M. A. Haidekker , C. R. White , and J. A. Frangos , J. Biomech. Eng. 123, 455 (2001). 10.1115/1.1389460 11601731

[26] D. N. Ku , D. P. Giddens , C. K. Zarins , and S. Glagov , Arterioscler., Thromb., Vasc. Biol. 5, 293 (1985). 10.1161/01.ATV.5.3.293

[27] M. Chachisvilis , Y.-L. Zhang , and J. A. Frangos , Proc. Natl. Acad. Sci. U. S. A. 103, 15463 (2006). 10.1073/pnas.0607224103 17030791 PMC1622845

[28] P. Oh and J. E. Schnitzer , Mol. Biol. Cell 12, 685 (2001). 10.1091/mbc.12.3.685 11251080 PMC30973

[29] M. A. Haidekker , N. L'Heureux , and J. A. Frangos , Am. J. Physiol. Heart Circ. Physiol. 278, H1401 (2000). 10.1152/ajpheart.2000.278.4.H1401 10749738

[30] A. Pierres , V. Monnet-Corti , A.-M. M. Benoliel , and P. Bongrand , Trends Cell Biol. 19, 428 (2009). 10.1016/j.tcb.2009.05.009 19709883

[31] P. J. Butler , T.-C. C. Tsou , J. Y.-S. Li , S. Usami , and S. Chien , FASEB J. 16, 216 (2002). 10.1096/fj.01-0434fje 11744620

[32] P. J. Butler , G. Norwich , S. Weinbaum , and S. Chien , Am. J. Physiol. Cell Physiol. 280, C962 (2001). 10.1152/ajpcell.2001.280.4.C962 11245613

[33] T. Tabouillot , H. S. Muddana , and P. J. Butler , Cell. Mol. Bioeng. 4, 169–181 (2011). 10.1007/s12195-010-0136-9 22247740 PMC3254098

[34] M. A. Haidekker , H. Y. Stevens , and J. A. Frangos , Ann. Biomed. Eng. 32, 531 (2004). 10.1023/B:ABME.0000019172.12700.b8 15117026

[35] S. R. Shaikh and M. A. Edidin , Chem. Phys. Lipids 144(1), 1 (2006). 10.1016/j.chemphyslip.2006.06.017 16945359

[36] F. Tokumasu , A. J. Jin , G. W. Feigenson , and J. A. Dvorak , Biophys. J. 84, 2609 (2003). 10.1016/S0006-3495(03)75066-8 12668469 PMC1302827

[37] N. Shamitko-Klingensmith , K. M. Molchanoff , K. A. Burke , G. J. Magnone , and J. Legleiter , Langmuir 28, 13411 (2012). 10.1021/la302705f 22924735

[38] K. Yamamoto and J. Ando , J. Cell Sci. 126, 1227 (2013). 10.1242/jcs.119628 23378020

[39] X.-B. Chen , L.-S. Niu , and H.-J. Shi , Biophys. Chem. 135, 84 (2008). 10.1016/j.bpc.2008.03.007 18440120

[40] S. Chien , Prog. Biophys. Mol. Biol. 83, 131 (2003). 10.1016/S0079-6107(03)00053-1 12865076

[41] S. Lu , M. Ouyang , J. Seong , J. Zhang , S. Chien , and Y. Wang , PLoS Comput. Biol. 4, e1000127 (2008). 10.1371/journal.pcbi.1000127 18711637 PMC2517613

[42] Y. Wang , H. Miao , S. Li , K.-D. Chen , Y.-S. Li , S. Yuan , J. Y.-J. Shyy , and S. Chien , Am. J. Physiol. Physiol. 283, C1540 (2002). 10.1152/ajpcell.00222.2002 12372815

[43] A. J. Maniotis , C. S. Chen , and D. E. Ingber , Proc. Natl. Acad. Sci. U. S. A. 94, 849 (1997). 10.1073/pnas.94.3.849 9023345 PMC19602

[44] B. P. Helmke , D. B. Thakker , R. D. Goldman , and P. F. Davies , Biophys. J. 80, 184 (2001). 10.1016/S0006-3495(01)76006-7 11159394 PMC1301225

[45] P. F. Davies , A. Robotewskyj , and M. L. Griem , J. Clin. Invest. 93, 2031 (1994). 10.1172/JCI117197 8182135 PMC294317

[46] M. C. Ferko , A. Bhatnagar , M. B. Garcia , and P. J. Butler , Ann. Biomed. Eng. 35, 858–859 (2007). 10.1007/s10439-007-9280-3 PMC3251212 17160699

[47] M. Dabagh , P. Jalali , P. J. P. J. Butler , and J. M. J. M. Tarbell , J. R. Soc. Interface 11, 20140431 (2014). 10.1098/rsif.2014.0431 24966239 PMC4233691

[48] M. Dabagh , P. Jalali , P. J. Butler , A. Randles , and J. M. Tarbell , J. R. Soc. Interface 14, 20170185 (2017). 10.1098/rsif.2017.0185 28515328 PMC5454307

[49] P. J. Butler and A. Bhatnagar , Biorheology 56(2–3), 101–112 (2019). 10.3233/BIR-190212 31561318

[50] N. Nakao , K. Maki , M. R. K. Mofrad , and T. Adachi , Biochem. Biophys. Res. Commun. 518, 579 (2019). 10.1016/j.bbrc.2019.08.091 31451222

[51] D. E. Fuentes , C. Bae , and P. J. Butler , Cell. Mol. Bioeng. 4, 616 (2011). 10.1007/s12195-011-0214-7 22247742 PMC3256556

[52] D. E. Fuentes and P. J. Butler , Cell. Mol. Bioeng. 5, 143 (2012). 10.1007/s12195-012-0225-z 23487555 PMC3593241

[53] C. G. Galbraith , K. M. Yamada , and J. A. Galbraith , Science 315, 992 (2007). 10.1126/science.1137904 17303755

[54] S. Son , G. J. Moroney , and P. J. Butler , Biophys. J. 113, 1080 (2017). 10.1016/j.bpj.2017.07.010 28877491 PMC5611673

[55] H. S. Muddana , H. H. Chiang , and P. J. Butler , Biophys. J. 102, 489 (2012). 10.1016/j.bpj.2011.12.033 22325271 PMC3274828

[56] F. Kong , A. J. García , A. P. Mould , M. J. Humphries , and C. Zhu , J. Cell Biol. 185, 1275 (2009). 10.1083/jcb.200810002 19564406 PMC2712956

[57] D. J. Dorahy and G. F. Burns , Biochem. J. 333, 373 (1998). 10.1042/bj3330373 9657978 PMC1219595

[58] C. Eggeling , C. Ringemann , R. Medda , G. Schwarzmann , K. Sandhoff , S. Polyakova , V. N. Belov , B. Hein , C. von Middendorff , A. Schönle , S. W. Hell , and A. Schonle , Nature 457, 1159 (2009). 10.1038/nature07596 19098897

[59] V. Mueller , C. Ringemann , A. Honigmann , G. Schwarzmann , R. Medda , M. Leutenegger , S. Polyakova , V. N. Belov , S. W. Hell , and C. Eggeling , Biophys. J. 101, 1651 (2011). 10.1016/j.bpj.2011.09.006 21961591 PMC3183802

[60] M. J. Paszek , D. Boettiger , V. M. Weaver , and D. A. Hammer , PLoS Comput. Biol. 5, e1000604 (2009). 10.1371/journal.pcbi.1000604 20011123 PMC2782178

[61] M. J. Paszek , C. C. DuFort , O. Rossier , R. Bainer , J. K. Mouw , K. Godula , J. E. Hudak , J. N. Lakins , A. C. Wijekoon , L. Cassereau , M. G. Rubashkin , M. J. Magbanua , K. S. Thorn , M. W. Davidson , H. S. Rugo , J. W. Park , D. A. Hammer , G. Giannone , C. R. Bertozzi , and V. M. Weaver , Nature 511, 319 (2014). 10.1038/nature13535 25030168 PMC4487551

[62] H. S. Muddana , S. Sengupta , T. E. Mallouk , A. Sen , and P. J. Butler , J. Am. Chem. Soc. 132, 2110 (2010). 10.1021/ja908773a 20108965 PMC2832858

[63] W. O. Hancock , Nat. Rev. Mol. Cell Biol. 15, 615 (2014). 10.1038/nrm3853 25118718 PMC5014371

[64] S. Sengupta , M. M. Spiering , K. K. Dey , W. Duan , D. Patra , P. J. Butler , R. D. Astumian , S. J. Benkovic , and A. Sen , ACS Nano 8, 140306092224001 (2014). 10.1021/nn405963x 24601532

[65] S. Ghosh , F. Mohajerani , S. Son , D. Velegol , P. J. Butler , and A. Sen , Nano Lett. 19, 6019 (2019). 10.1021/acs.nanolett.9b01830 31429577

[66] Y. Park , C. A. Best , T. Auth , N. S. Gov , S. A. Safran , G. Popescu , S. Suresh , and M. S. Feld , Proc. Natl. Acad. Sci. U. S. A. 107, 1289 (2010). 10.1073/pnas.0910785107 20080583 PMC2802590

[67] T. Betz , M. Lenz , J. F. Joanny , and C. Sykes , Proc. Natl. Acad. Sci. U. S. A. 106, 15320 (2009). 10.1073/pnas.0904614106 19717437 PMC2741249

[68] K. K. Dey , S. Das , M. F. Poyton , S. Sengupta , P. J. Butler , P. S. Cremer , and A. Sen , ACS Nano 8, 11941 (2014). 10.1021/nn504418u 25243599

[69] S. An , R. Kumar , E. D. Sheets , and S. J. Benkovic , Science 320, 103 (2008). 10.1126/science.1152241 18388293

[70] X. Zhao , H. Palacci , V. Yadav , M. M. Spiering , M. K. Gilson , P. J. Butler , H. Hess , S. J. Benkovic , and A. Sen , Nat. Chem. 10, 311 (2017). 29461522 10.1038/nchem.2905

[71] J. H. Dangaria and P. J. Butler , Am. J. Physiol. Cell Physiol. 293, C1568 (2007). 10.1152/ajpcell.00193.2007 17670893 PMC3251213

[72] R. Krishnan , C. Y. Park , Y.-C. Lin , J. Mead , R. T. Jaspers , X. Trepat , G. Lenormand , D. Tambe , A. V. Smolensky , A. H. Knoll , J. P. Butler , and J. J. Fredberg , PLoS One 4, e5486 (2009). 10.1371/journal.pone.0005486 19424501 PMC2675060

[73] E. a Osborn , A. Rabodzey , C. F. Dewey , and J. H. Hartwig , Am. J. Physiol. Cell Physiol. 290, C444 (2006). 10.1152/ajpcell.00218.2005 16176968

[74] G. A. Clawson , T. Abraham , W. Pan , X. Tang , S. S. Linton , C. O. McGovern , W. S. Loc , J. P. Smith , P. J. Butler , M. Kester , J. H. Adair , and G. L. Matters , Nucleic Acid Ther. 27, 23 (2017). 10.1089/nat.2016.0621 27754762 PMC5312616

[75] B. Lan , R. Krishnan , C. Y. Park , R. A. Watanabe , R. Panganiban , J. P. Butler , Q. Lu , W. C. Cole , and J. J. Fredberg , Am. J. Physiol. Cell. Mol. Physiol. 314, L799 (2018). 10.1152/ajplung.00326.2017 PMC6008132 29345194

[76] T. Wu and J. J. Feng , Biophys. J. 108, 43 (2015). 10.1016/j.bpj.2014.11.015 25564851 PMC4286609

[77] R. Krishnan , E. P. Canović , A. L. Iordan , K. Rajendran , G. Manomohan , A. P. Pirentis , M. L. Smith , J. P. Butler , J. J. Fredberg , and D. Stamenović , Am. J. Physiol. Physiol. 303, C368 (2012). 10.1152/ajpcell.00074.2012 PMC3422985 22700796

[78] M. Collins , F. Mohajerani , S. Ghosh , R. Guha , T.-H. Lee , P. J. Butler , A. Sen , and D. Velegol , ACS Nano 13, 8946 (2019). 10.1021/acsnano.9b02811 31291087

[79] P. J. Butler , K. K. Dey , and A. Sen , Cell. Mol. Bioeng. 8, 106–118 (2015). 10.1007/s12195-014-0376-1 26019728 PMC4442092

[80] V. Yadav , W. Duan , P. J. Butler , and A. Sen , Annu. Rev. Biophys. 44, 77 (2015). 10.1146/annurev-biophys-060414-034216 26098511

[81] S. Chien , Ann. Biomed. Eng. 36, 554 (2008). 10.1007/s10439-007-9426-3 18172767 PMC3718045

[82] W. B. Cannon , *The Wisdom of the Body* ( W.W. Norton and Co, 1932).

[83] B. D. Hoffman , G. Massiera , K. M. Van Citters , and J. C. Crocker , Proc. Natl. Acad. Sci. U. S. A. 103, 10259 (2006). 10.1073/pnas.0510348103 16793927 PMC1502445

[84] E. Sezgin , F. Schneider , S. Galiani , I. Urbančič , D. Waithe , B. C. Lagerholm , and C. Eggeling , Nat. Protoc. 14, 1054 (2019). 10.1038/s41596-019-0127-9 30842616

[85] S. Culley , K. L. Tosheva , P. M. Pereira , and R. Henriques , Int. J. Biochem. Cell Biol. 101, 74 (2018). 10.1016/j.biocel.2018.05.014 29852248 PMC6025290

[86] M. Amodio , D. van Dijk , K. Srinivasan , W. S. Chen , H. Mohsen , K. R. Moon , A. Campbell , Y. Zhao , X. Wang , M. Venkataswamy , A. Desai , V. Ravi , P. Kumar , R. Montgomery , G. Wolf , and S. Krishnaswamy , Nat. Methods 16, 1139–1145 (2019). 10.1038/s41592-019-0576-7 31591579 PMC10164410

[87] T. Sanchez , D. T. N. Chen , S. J. DeCamp , M. Heymann , and Z. Dogic , Nature 491, 431 (2012). 10.1038/nature11591 23135402 PMC3499644

[88] F. M. Pavalko , S. M. Norvell , D. B. Burr , C. H. Turner , R. L. Duncan , and J. P. Bidwell , J. Cell. Biochem. 88, 104 (2003). 10.1002/jcb.10284 12461779

[89] J. P. Bidwell and F. M. Pavalko , Sci. Signal. 3, pe51 (2010). 10.1126/scisignal.3153pe51 21177492

[90] R. G. Parton and M. A. del Pozo , Nat. Rev. Mol. Cell Biol. 14, 98 (2013). 10.1038/nrm3512 23340574

